# Hp1404, a New Antimicrobial Peptide from the Scorpion *Heterometrus petersii*

**DOI:** 10.1371/journal.pone.0097539

**Published:** 2014-05-14

**Authors:** Zhongjie Li, Xiaobo Xu, Lanxia Meng, Qian Zhang, Luyang Cao, Wenxin Li, Yingliang Wu, Zhijian Cao

**Affiliations:** State Key Laboratory of Virology, College of Life Sciences, Wuhan University, Wuhan, PR China; Faculdade de Medicine da Universidade de Lisboa, Portugal

## Abstract

Antimicrobial peptides have attracted much interest as a novel class of antibiotics against a variety of microbes including antibiotics resistant strains. In this study, a new cationic antimicrobial peptide Hp1404 was identified from the scorpion *Heterometrus petersii*, which is an amphipathic α-helical peptide and has a specific inhibitory activity against gram-positive bacteria including methicillin-resistant *Staphylococcus aureus*. Hp1404 can penetrate the membrane of *S. aureus* at low concentration, and disrupts the cellular membrane directly at super high concentration. *S. aureus* does not develop drug resistance after multiple treatments with Hp1404 at sub MIC concentration, which is possibly associated with the antibacterial mechanism of the peptide. In addition, Hp1404 has low toxicity to both mammalian cells (HC_50_  =  226.6 µg/mL and CC_50_ > 100 µg/mL) and balb-c mice (Non-toxicity at 80 mg/Kg by intraperitoneal injection and LD_50_  =  89.8 mg/Kg by intravenous injection). Interestingly, Hp1404 can improve the survival rate of the MRSA infected balb-c mice in the peritonitis model. Taken together, Hp1404 may have potential applications as an antibacterial agent.

## Introduction

The increasing frequency of antibiotic resistance among microorganisms is becoming a more and more serious problem, which has outpaced the development of new antibiotics [1], [2], [3]. It is urgently needed to discover new and more effective antimicrobial agents. As a potential source of these agents, antimicrobial peptide (AMPs) are ubiquitous in nature, which can be found in microorganisms [4], insects [5], amphibians [6], mammals [7], and plants [8]. They are produced as a part of the innate immune system defense, and show potent antimicrobial activity against a broad spectrum of microorganisms including resistant strains [9]. Interestingly, the mechanisms of AMPs's action are different from conventional antibiotics, most of which kill microorganisms rapidly by disrupting the integrity of the cytoplasmic membrane [10], [11]. Some of them can also interfere with the intracellular processes, such as affecting cell-wall biosynthesis pathway, inhibiting protein biosynthesis, or interacting with nucleic acids [12]. These properties make them the attractive candidates for the development of new antimicrobial agents in overcoming microbial resistance. At least 2300 different AMPs have been studied (http://aps.unmc.edu/AP/main.php) during the last three decades, and several AMPs have been investigated as therapeutic agents in the past decade [9].

As a living fossil, scorpion has survived over 400 million years on earth, and developed diverse venom peptides for successful survival during its long-term evolution [13]. So far, more and more AMPs have been identified from scorpion venoms, which can be divided into disulphide-bridged and non-disulphide-bridged peptides. Scorpine, a triple disulphide-bridge AMP from the scorpion *Pandinus imperator* has anti-bacterial and anti-malaria activities [14]. Non-disulphide-bridged AMPs Pandinins (from the scorpion *Pandinus imperator*) and IsCTs (from the scorpion *Opisthacanthus madagascariensis*) are α-helical polycationic peptides and have antimicrobial activity against both gram-positive bacteria and gram-negative bacteria [15], [16], [17]. The non-disulphide-bridged AMP Vejovine from the scorpion *Vaejovis mexicanus* can inhibit the growth of multidrug resistant clinical isolates of gram-negative bacteria [18]. These findings make scorpion venom as a potential source for discovering AMPs. We focus our interest on the scorpion species *Heterometrus petersii*, which usually inhabits in tropical to subtropical rainforests. Various kinds of bacteria can grow and proliferate in this kind of living environment, which is conducive to the evolution of the scorpion venom to contain more AMPs.

In this study, a new AMP named Hp1404 was characterized from the venomous gland cDNA library of the scorpion *Heterometrus petersii*. Hp1404 is an amphipathic α-helical peptide. The *in vitro* antibacterial activities of Hp1404 peptide were then investigated using both standard and resistant strains. The mechanism of Hp1404 against bacteria was further explored in our work. Finally, we tested the toxicities of Hp1404 against mammalian cells and mice and the protective effect of Hp1404 against infection to evaluate its potential application as an antibacterial agent.

## Materials and Methods

### Ethics statement

The scorpion *Heterometrus petersii* used in this work was obtained from a scorpion breeding base in Hubei, province of China. The female balb-c (18–21 g) mice were obtained from the Animal Facility at Wuhan University Zhong Nan Hospital. The mice were maintained under standard conditions of humidity (50 ± 5%), temperature (25 ± 2°C) and dark and light cycles (12 h each) with free access to food and water. When met certain clinical criteria or at the end of the experiments, the mice were humanely euthanized (anesthetized by intra-peritoneal injection of pentobarbital, and sacrifice by cervical dislocation). All animal experiments were approved by the Institutional Animal Care and Use Committee of Wuhan University. The fresh human red blood cells (hBRCs) were from healthy donor Zhongjie Li, who is the author of this manuscript. The hBRCs-related experiment was approved by the Ethics Committee of the College of Life Sciences of Wuhan University.

### cDNA library construction and sequencing

Twenty specimens of *H. petersii* were collected and milked for 2 days by electrical stimulation. Total RNA was prepared from the glands by using TRIzol reagent (Invitrogen). Poly (A) mRNA was purified by using a Poly (A) Tract mRNA isolation system (Promega). The cDNA library was constructed with the Superscript plasmid system cDNA library construction kit (Gibco/BRL). cDNAs were cloned into the pSPORT1 plasmid (Gibco/BRL) and transformed into *Escherichia coli* DH5α cells. cDNA clones were randomly chosen and sequenced to obtain a reliable representation of the toxin content in the venom gland. Positive clones were identified by using an ABI Prism 377XL DNA sequencer with a universal T7 promoter primer.

### Sequence and secondary structure analysis

Sequence analysis was carried out by using BLASTX (http://blast.ncbi.nlm.nih.gov/Blast.cgi), DNAMAN, and GENRUNR. The secondary structure was analyzed by using the online program Heliquest (http://heliquest.ipmc.cnrs.fr/cgi-bin/ComputParams.py) and measured by circular dichroism (CD) spectroscopy. The CD assay was performed at room temperature at the UV range of 190–250 nm at a concentration of 0.1 mg/mL in: (i) water, (ii) 30% TFE/H_2_O, or (iii) 70% TFE/H_2_O. Spectra were collected from three separate recordings.

### Peptide synthesis and bacterial strains

The peptides used in this study were synthesized by GL Biochem (Shanghai, China) with amidated C-terminus, and with a purity of >95%. *Staphylococcus aureus* AB94004, *S. aureus* ATCC25923, *S. aureus* ATCC6538, *S. aureus* AB208193, *S. epidermidis* AB208187, *S. epidermidis* AB208188, *Micrococcus luteus* AB93113, *Bacillus subtilis* AB91021, *E. coli* AB94012, *E. coli* ATCC25922, *Pseudomonas aeruginosa* AB93066, *Pseudomonas aeruginosa* ATCC9027 and *Pseudomonas aeruginosa* ATCC27853 were purchased from the China Center of Type Culture Collection (CCTCC). MRSA P1381 and MRSA P1374 were obtained from the 302^nd^ military hospital of Beijing, China. *Enterococcus faecium*, *Streptococcus agalactiae* were clinical strains from a hospital.

### Antimicrobial assays

The antimicrobial activity was determined in the broth microdilution assay from the procedure recommended by the Clinical and Laboratory Standards Institute with some modifications. Briefly, bacteria were cultured in Luria-Bertani (LB) medium to OD_600_  =  0.6 at 37 °C, then diluted to 10^5^–10^6^ cfu/mL in LB medium, and peptide was serially diluted in 0.9% saline. 160 µL of the bacterial suspension and 40 µL of peptide dilution at varying concentrations were added into 96-well plates, and then incubated for 16 h at 37 °C with continuous shaking at 250 rpm. The minimum inhibitory concentration (MIC) was determined as the lowest peptide concentration at which no bacterial growth was observed.

### Competition assays

A total of 1 mg/mL of Hp1404 peptide solution was mixed with an equal volume of 5 mg/mL of the lipoteichoic acid (LTA, sigma-aldrich: L2515) or lipopolysaccharide (LPS, sigma-aldrich: L3012) solution. Then, the MICs of Hp1404 treated with LTA or LPS against *S. aureus* AB94004 were measured. The experiment was verified by three independent trials.

### Time-killing kinetics

*S. aureus* AB94004 was cultured in LB medium to exponential phase (OD_600_  =  0.5), then diluted to ca. 10^7^ cfu/mL in LB medium, and treated with different concentrations of peptide solutions. Aliquots were taken at defined intervals and washed with 0.9% saline, and then diluted appropriately in the saline and plated on LB agar. The plates were incubated at 37 °C for 24 h, and the colony forming units (CFU) were counted.

### Confocal laser-scanning microscopy

The assay was measured according to the method [19] with some modifications. *S. aureus* AB94004 was cultured to mid logarithmic phase (ca. 10^7^ cfu/mL), harvested by centrifugation, and washed and resuspended with 15 mM sodium phosphates buffer (pH 7.4). After incubated with 12.5 µg/mL fluorescein isothiocyanate (FITC)-labeled peptide at 37 °C for 20 min, the cells were washed. The glass slide was immersed in the suspension for 10 min to let the cells immobilized, and then examined by an Olympus IX70 confocal laser-scanning microscope.

### Transmission electron microscopy (TEM)

The exponential phase bacteria *S. aureus* AB94004 (ca. 10^7^ cfu/mL) was incubated with Hp1404 at the final concentration of 2 × MIC or 5 × MIC or without Hp1404 for 20 min at 37 °C. Samples were harvested by centrifugation, and washed with 0.9% salt solution. The bacteria were fixed with 2% glutaraldehyde in 0.1 M PBS for 1 h, and with 1% osmium tetroxide at 4 °C for another 1 h. After fixation, the bacteria were stained with 1% uranyl acetate and then dehydrated by using a series of graded ethyl alcohols. After this, the samples were embedded in epoxy resins and stained with 1% uranyl acetate and lead citrate. The samples were then semi-thin sectioned and examined by using a HITACHIH-8100 transmission electron microscope.

### Fluorescence measurements

*S. aureus* AB94004 was cultured to exponential phase (ca. 10^7^ cfu/mL), then the fluorescence dye SYTOX green (Invitrogen) was added at a final concentration of 5 µM. After incubated for 10 min, the peptides were added at a final concentration of 1 × MIC, 2 × MIC. Fluorescence was measured at the excitation and emission wavelengths of 488 and 525 nm, respectively.

### Drug resistant assays

The initial MIC of Hp1404 and control antibiotics kanamycin against *S.aureus* AB94004 was obtained as described above. Bacteria from duplicate wells at the concentration of 1/2-MIC were then diluted to 10^5^∼10^6^ cfu/mL in LB medium for the new MIC assay. The experiment was repeated each day for 15 passages.

### Checkerboard assay

The checkerboard assay is widely used for evaluation of properties of different antibacterial combinations [20]. Kanamycin was serially diluted in 0.9% saline in the presence of a constant amount of peptide, equal to one-quarter of the peptide MIC. Then the combination MIC was measured. The fractional inhibitory concentration index (FICI) was used to determine the synergy between antimicrobial agents. Synergy was defined as an FICI ≤ 0.5. Indifference or absence of interaction was defined as 0.5 < FICI < 4. Antagonism was defined as FICI > 4.The FICI was calculated as follows: 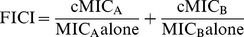, cMIC: MIC in combination.

### Hemolytic activity

Fresh hRBCs from healthy donors were washed 3 times in 0.9% saline, and resuspended in the same salt solution to a concentration of 2% (v/v). 100 µL of peptide solution and 100 µL of RBC suspension were added to the wells of 96-well plate, which made the final concentration of hBRCs was 1% (v/v). After incubated for 1 h at 37 °C with gently shaking, samples were centrifuged at 1000 g for 10 min. The supernatant (100 µL) from each well was transferred to a new 96-well plate and the absorbance was measured at 490 nm. 0.9% saline and 1% Triton X-100 was served as negative and positive controls, respectively. 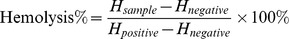. *H*: absorbance at 490 nm.

### Cell culture and MTT assay

The HFF, HEK293T and A375 cell lines were cultured in Dulbecco's modified Eagle medium supplemented with 100 µg/mL streptomycin, 100 U/mL penicillin and 10% (v/v) fetal bovine serum, and maintained in a humidified chamber with 5% CO_2_ at 37 °C. Cytotoxicity of Hp1404 against the mammalian cells was evaluated by MTT assays. Cells (6000 cells/well) were pre-seeded on sterilized 96-well plates in 100 µL medium and incubated for 24 h. Dilutions of peptides in the same medium (100 µL) were added and incubated for another 24 h. Then 20 µL of 3-(4,5-dimethylthiazol-2yl)-2,5-diphenyltetrazolium bromide (MTT) solution (5 mg/mL) was added to each well and the plates were incubated for an additional 4 h. The supernatant was removed, and 100 µL dimethyl sulfoxide was added to the wells to dissolve any remaining precipitate. Absorbance at 570 nm was measured using an ELISA reader. No peptide and 0.1% Triton X-100 was served as negative and positive controls, respectively. The viability percentage was calculated according to the equation: 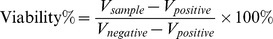. *V*: absorbance at 570 nm.

### Acute toxicity

The acute systemic toxicity of Hp1404 to mice was evaluated by the determination of the LD_50_ (50% lethal dose). After being dissolved in 0.9% saline, Hp1404 was given to female balb-c mice (18–21 g) by intraperitoneal (i.p.) or intravenous (i.v.) injection at the designated doses in 0.4 mL, six mice per group. The number of mice surviving at each group was monitored for up to a period of 7 days after treatment, and the values of LD_50_ were calculated by the Karber method [21].

### Mouse peritonitis model

The mouse peritonitis model was based on a previously described protocol [22]. Female balb-c mice (18–21 g) were infected by i.p. injection of 0.5 mL of the MRSA P1381 inoculum (10^8^ CFU) in 0.9% saline containing 5% (wt/vol) mucin. One hour after bacterial administration, mice (6 mice per group) were treated with a single dose of peptide by (i) i.p. or (ii) i.v. injection. Vancomycin and 0.9% saline treatment were used as positive and negative controls, respectively. The mice were observed 2–4 times daily for 7 days. The mice were euthanized if they became moribund (unresponsive to external stimuli, can not swing the neck, and can not eat and drink), and counted as non-survivor.

## Results

### Characterization and analysis of Hp1404

**Figure 1:**
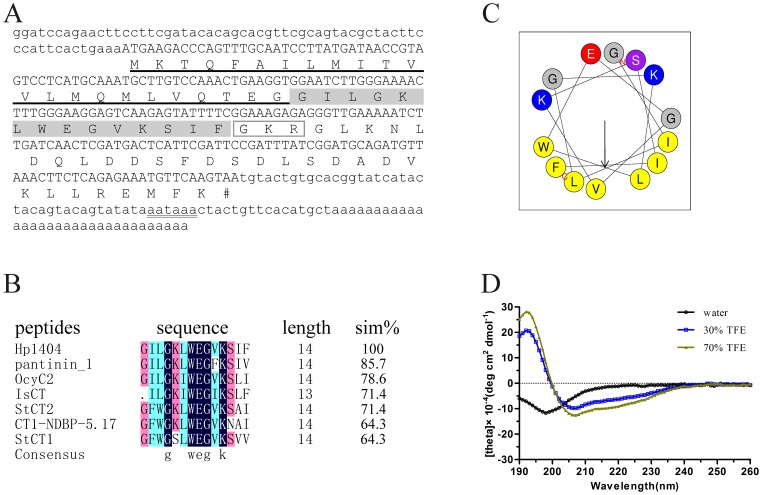
Sequence analysis, multiple alignment, and secondary structure analysis of Hp1404 peptide. (A) cDNA and deduced amino acid sequences of Hp1404.The deduced amino acid residues are shown below the corresponding nucleotide sequences. The signal peptide residues are underlined. The mature peptide residues are shaded in gray.The potential cleavage site for prohormone convertase is marked in rectangle. The potential polyadenylation signal aataaa is double underlined. (B) Multiple alignment of Hp1404 with other antimicrobial peptides. Sim%, the percentage of sequence identity relative to Hp1404. The different color represents the different homology level. StCT1 and StCT2 from the scorpion *Scorpiops tibetanus*, CT1-NDBP-5.17 from the scorpion *Urodacus yaschenkoi*, IsCT from the scorpion *Opisthacanthus madagascariensis*, OcyC2 from the scorpion *Opisthacanthus cayaporum*, pantinin 1 from the scorpion *Pandinus imperator*. (C) Helical wheel diagram of Hp1404 determined by the Heliquest method. The representation of Hp1404 as a helical wheel shows the hydrophilic face and hydrophobic face. (D) CD spectra of Hp1404 peptide (100 µg/mL) in water alone or with 30 or 70% aqueous TFE.

After systemic screening the clones from the venom gland cDNA library of the scorpion *H. petersii*, we obtained a new peptide precursor named Hp1404. As shown in Figure 1A, the cDNA sequence of Hp1404 consists of a 5′ UTR of 62 nt, an ORF of 210 nt, and a 3′ UTR of 92 nt. A single polyadenylation signal (AATAAA) was found 16 nt upstream of the poly (A) tail. The ORF of 210 nt encodes a precursor that consists of 69 amino acid residues, which contains a putative 23-residue signal peptide (http://www.cbs.dtu.dk/services/SignalP/) and a 46-residue propeptide. According to previous studies [23], [24], further bioinformatic analysis indicated that the propeptide starts with a conserved posttranslational processing signal Gly-Lys-Arg at positions 38 to 40, and would result in a 14-residue mature peptide (GILGKLWEGVKSIF) with C-terminal amidation.

Multiple sequence alignment revealed that Hp1404 shared a high identity (Figure 1B) with the AMPs StCT1 [25], CT1-NDBP-5.17 [26], IsCT [27], StCT2 [28], OcyC2 [29], and pantinin 1 [30], which suggests that Hp1404 peptide may have antibacterial activity.

Secondary structure predicted by using the online program Heliquest (http://heliquest.ipmc.cnrs.fr/cgi-bin/ComputParams.py) indicated that Hp1404 was a typical amphipathic α-helix, the helical wheel is divided into two parts: one part is the hydrophobic face, and the other is the hydrophilic face (Figure 1C). Such structure was further confirmed by the CD spectral analysis. As shown in Figure 1D, Hp1404 exhibited only a large negative peak at 198 nm in water, indicating a random coil structure, while Hp1404 exhibited a large positive peak at around 195 nm and large negative bands at 208 and 220 nm in TFE, indicating predominance of α-helices [31], [32]. These results suggested that Hp1404 could form an amphipathic helical structure in the appropriate membrane environment.

### Antimicrobial activity *in vitro*

As shown in Table 1, Hp1404 has anti-bacterial activity with MICs of 6.25–25 µg/mL against gram-positive bacteria including MRSA and other clinical strains, but it can not inhibit the growth of gram-negative bacteria at the concentration of 100 µg/mL.

**Table 1:** MICs of Hp1404 against bacteria.

Strains	MIC	
	μg/mL	μM
**Gram-positive**		
*Staphylococcus aureus* AB94004	12.5	8.08
*Staphylococcus aureus* ATCC25923	6.25	4.04
*Staphylococcus aureus* ATCC6538	12.5	8.08
*Staphylococcus aureus* AB208193	12.5	8.08
*Staphylococcus epidermidis* AB208187	25	16.16
*Staphylococcus epidermidis* AB208188	25	16.16
*Micrococcus luteus* AB93113	12.5	8.08
*Bacillus subtilis* AB91021	12.5	8.08
*Staphylococcus aureus* MRSA P1381	12.5	8.08
*Staphylococcus aureus* MRSA P1374	25	16.16
*Enterococcus faecium*	12.5	8.08
*Streptococcus agalactiae*	25	16.16
**Gram-negative**		
*Escherichia coli* AB94012	> 100	> 64.6
*Escherichia coli* ATCC25922	> 100	> 64.6
*Pseudomonas aeruginosa* AB93066	> 100	> 64.6
*Pseudomonas aeruginosa* ATCC9027	> 100	> 64.6
*Pseudomonas aeruginosa* ATCC27853	> 100	> 64.6

### LTA and LPS competition assays

To determine whether LTA or LPS interfere with the antimicrobial activity of Hp1404, MICs of Hp1404 mixed with LTA or LPS against *S. aureus* were measured. Our results (data not shown) showed that the MICs of Hp1404 treated with LTA or LPS were 12.5 µg/mL, which were the same as the untreated Hp1404.

### Antibacterial mechanism

**Figure 2:**
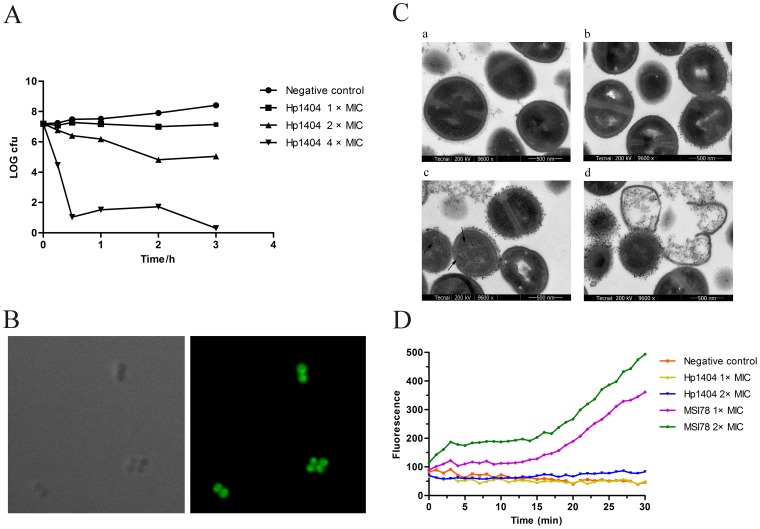
Antibacterial mechanism of the peptide Hp1404. (A) Time-kill kinetics of Hp1404 against *S. aureus* AB94004.The assay was performed by determining the counts of surviving bacteria, 0 h represents bacteria before treated. (B) Confocal fluorescence microscopic images of *S. aureus* treated with FITC-Hp1404. Left: normal image; right: fluorescence image. (C) Transmission electron microscopy of *S. aureus* treated with Hp1404. a: negative control. b: treated with Hp1404 at the concentration of 1×MIC for 20 min. c-d: treated with Hp1404 at the concentration of 5×MIC for 20 min. (D) Fluorescence measurements. Negative control, 0.9% saline. MSI78 is an amphipathic α-helical peptide with an antibacterial mechanism of disrupting the membrane. The MIC of MSI78 against *S. aureus* AB94004 is 6.25 µg/mL.

To gain insights into the antibacterial mechanism of Hp1404, *S. aureus* AB94004 was selected as the model bacteria. Firstly, the time-killing kinetics was studied. As shown in Figure 2A, the killing rate increased along with the increasing of the peptide concentration. The viable colony number only decreased 15% in 1 h at the concentration of 2 × MIC, but it decreased approximately 85% in 30 min at the concentration of 4 × MIC.

To explore the target site of Hp1404 in *S. aureus* AB94004, the distribution of Hp1404 in bacteria was investigated by confocal laser-scanning microscopy. As shown in Figure 2B, FITC-labeled Hp1404 penetrated the bacterial cell membrane and accumulated in the cytoplasm.

To determine the direct influence of Hp1404 on the bacteria, TEM was used to examine the ultrastructure changes of the bacteria. As shown in Figure 2C-a, untreated cells of *S. aureus* were round, proliferating cell with intact cell wall and well-defined membrane, and the intracellular DNA region displayed a heterogeneous electron density. At the concentration of 2 × MIC, the cell wall and membrane had no changes (Figure 2C-b). But, special structures could be observed in the cytoplasm (Figure 2C-c: marked by arrow) and some cells were completely lysed (Figure 2C-d) at the concentration of 5 × MIC.

To further assess whether Hp1404 could influence the integrity of the membrane, the ability of permeabilizing the cell membrane of Hp1404 was also tested with the fluorescent nucleic acid stain SYTOX. As shown in Figure 2D, no fluorescence increase was apparent over 30 min when *S. aureus* cells were exposed to SYTOX and Hp1404, compared with a rapid fluorescence increase upon exposure to MSI78, which is an amphipathic α-helical peptide with a antibacterial mechanism of disrupting the membrane [33].

### Drug resistant assay

**Figure 3:**
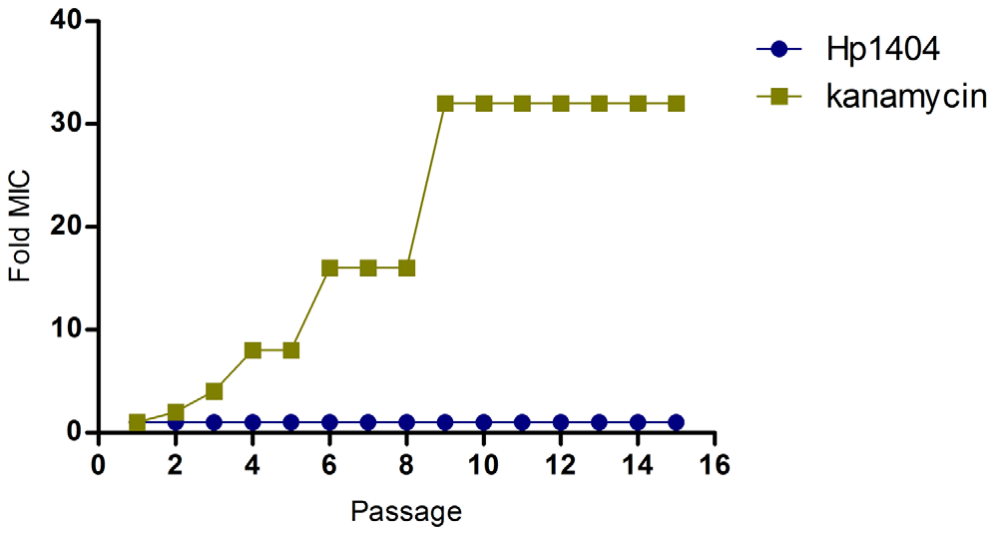
Resistance development of *S. aureus* AB94004 treated by Hp1404 or kanamycin.

The emergence of drug resistance in bacteria for conventional antibiotic treatments is a major public-health concern. The development of resistance to Hp1404 was evaluated in *S. aureus* AB94004. As shown in Figure 3, the multiple treatments with kanamycin (MIC: 6.25 µg/mL) gave rise to drug resistance as early as passage 2, and the MIC increased about 32-fold after the course of 9 passages, while there was no change in the MIC for Hp1404 during the course of 15 passages. These results indicated that it is difficult for *S. aureus* to develop resistance to Hp1404.

### Checkerboard assay

To evaluate whether Hp1404 exhibits synergistic effects with kanamycin, the checkerboard assay was performed. Our results showed that the fractional inhibitory concentration index was 0.5, which demonstrated that Hp1404 exhibited synergistic effects with kanamycin.

### *In vitro* toxicity

**Figure 4:**
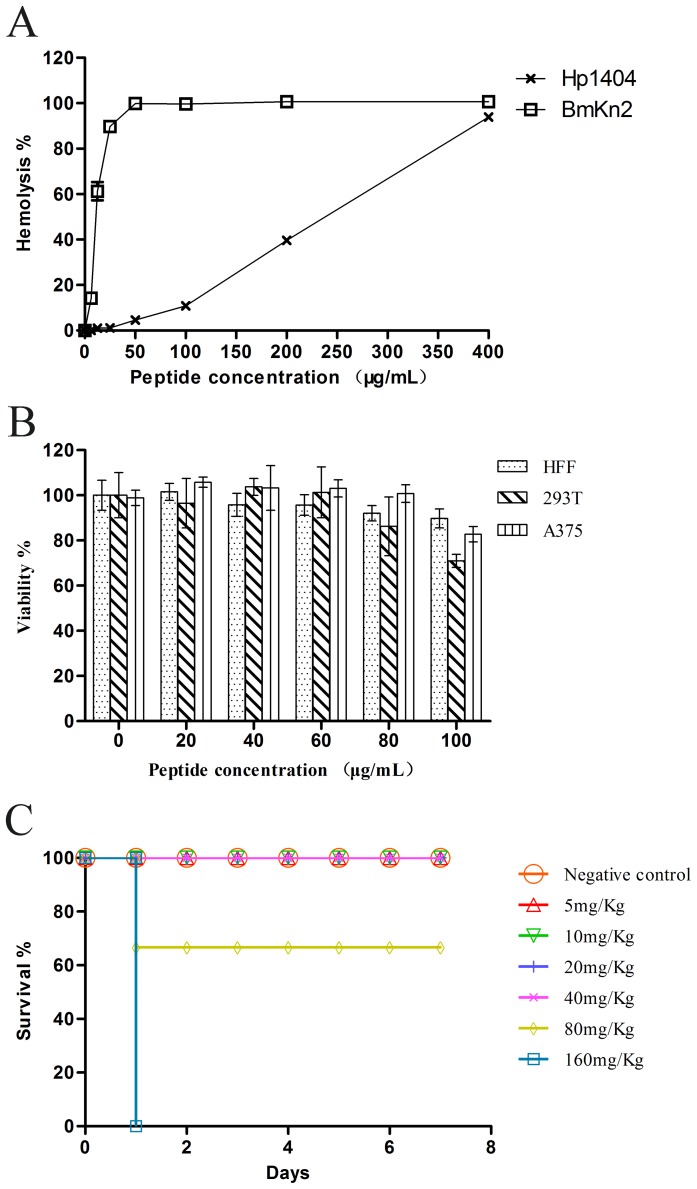
Toxicity of Hp1404 *in vitro* and *in vivo*. (A) Hemolytic activity of Hp1404 against human red blood cells. BmKn2 is a highly hemolytic antimicrobial peptide from the scorpion *Mesobuthus martensii Karsch*. (B) Cytotoxicities of Hp1404 against HFF, HEK293T and A375 cell lines. Cytotoxicity was measured by MTT assay. (C) Acute toxicity of Hp1404 to mice by intravenous injection.

In order to assess the toxicity of the peptide, the hemolytic activity to hRBCs and cytotoxicity to mammalian cell lines were tested. In comparison with BmKn2 [34], a highly hemolytic peptide, Hp1404 has a low hemolytic activity (about 10%) at the concentration 100 µg/mL (Figure 4A), and the HC_50_ (50% hemolytic concentration) of the peptide Hp1404 is 226.6 µg/mL (146.5 µM). The MTT tests showed that the peptide only has about 10–30% cytotoxicity to the cells be tested at the concentration of 100 µg/mL (Figure 4B), and the CC_50_ (50% cytotoxic concentration) is > 100 µg/mL (64.6 µM). Taken together, the toxicity of Hp1404 to the mammalian cells is lower than that to the sensitive bacteria.

### *In vivo* toxicity

Acute toxicity was examined to evaluate the in vivo toxicity of Hp1404. With the dose up to 80 mg/Kg, the mice injected with Hp1404 by i.p showed no immediate adverse events, and all the treated mice survived in the 7-day study period (data not shown). In the i.v. injection treatment, the mice injected with Hp1404 also showed no immediate adverse events at the dose of 5 or 10 mg/Kg, but had a 33.3% (2/6 mice) mortality at the dose of 80 mg/Kg and 100% (6/6 mice) mortality at the dose of 160 mg/Kg (Figure 4C). Thus, the LD_50_ of Hp1404 to mice is 89.8 mg/Kg by i.v. injection.

### *In vivo* antibacterial activity

**Figure 5:**
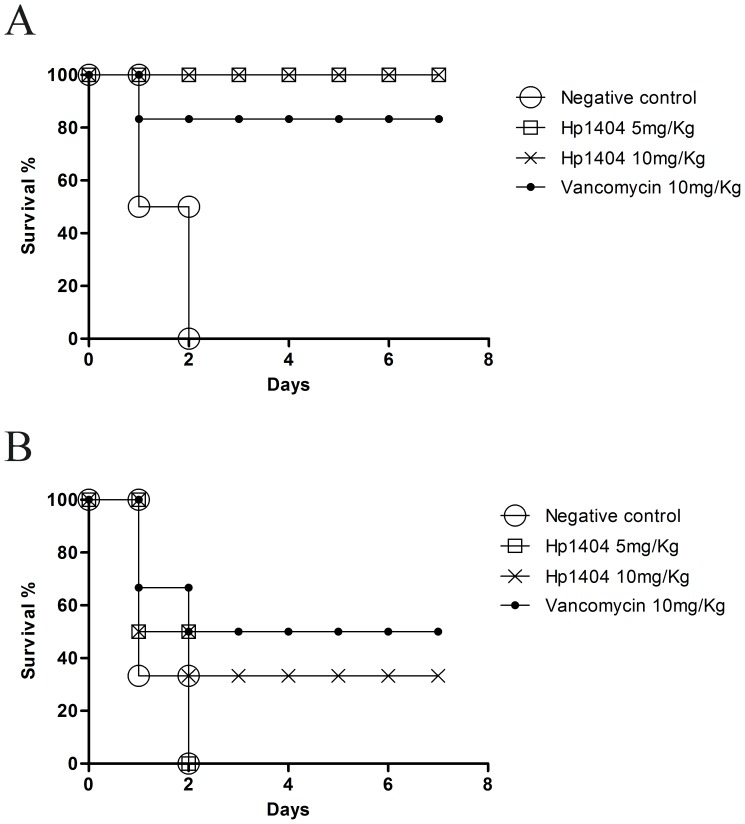
Antibacterial activity of Hp1404 *in vivo*. (A) Therapeutic efficacy of Hp1404 on MRSA infected mice by intraperitoneal injection. (B) Therapeutic efficacy of Hp1404 on MRSA infected mice by intravenous injection.

To further investigate the antibacterial activity of Hp1404 peptide *in vivo*, the potential therapeutic efficacy of the peptide Hp1404 was evaluated by a mouse peritonitis model. As shown in Figure 5A, all six mice in the negative control group died after inoculated with MRSA P1381 for 48 h, but the mice treated with the dose of 5 or 10 mg/Kg Hp1404 by i.p. injection showed a survival rate of 100%. On the other hand, the mice treated with the dose of 10 mg/Kg Hp1404 by i.v. injection also showed a survival rate of 33.3% (2/6 mice) in the 7-day study period (Figure 5B), but it was not as effective as the dose of 5 mg/Kg by i.p. All these data indicate that the peptide Hp1404 still has anti-bacterial activity *in vivo*.

## Discussion

In the present study, we indentified a new cationic AMP Hp1404 with a net charge of +1 from the scorpion *H. petersii*. In contrast to conventional AMPs with wide spectra of activity, Hp1404 just has a specific potent antimicrobial activity against gram-positive bacteria (Table 1). Previous studies showed that the interaction between AMPs and LPS or LTA is important to the activity of AMPs [12]. Our results showed that the antibacterial activity of Hp1404 was not interfered with LTA or LPS, which was different from the peptide Kn2–7, a scorpion AMP derivative studied in our previous work [35]. It implied that LPS or LTA might not be the primary antimicrobial action site of Hp1404. Our further data by biolayer interferometry experiments showed that Hp1404 did not interact with LPS or LTA indeed (data not shown). Thus, the mode of action of Hp1404 may be different from that of most classical AMPs [35]. Though Hp1404 do not interact with LTA, two factors may let it successful reach the action sites and perform its activity against gram-positive bacteria: (i) The structure of the gram-positive bacteria cell wall. Such as *S. aureus* cell, which has a loosely arrayed network on the cell surface, consisting of fibrils and pores ranging in size from 50 to 500 Å [36], [37], while the Hp1404 molecule (14aa) is about 20–50 Å. These pores are large enough to let the peptide molecules to pass through them. (ii) Hp1404 is a cationic peptide, while there are a lot of negatively charged molecules outside the bacterial cell, the electrostatic bonding between Hp1404 and bacteria will encourage the cationic peptide to pass through the cell wall [11].

Structure analysis suggested that Hp1404 was an amphipathic and α-helical peptide (Figure 1C, 1D). Usually, natural AMPs with such structure are considered to be membrane-lytic peptides [38], [39], which kill bacteria in 2 to 3 min after initial exposure [40], [41]. The killing of *S. aureus* by Hp1404 peptide was a rather slow process (Figure 2A), which suggested that Hp1404 might not be a classical membrane-lytic peptide. Such assumption was confirmed by TEM, which showed that the membrane was not lysed by Hp1404 at low MIC concentration (Figure 2C-b). Our Fluorescence measurements also showed that Hp1404 did not disrupt the membrane at low MIC concentration (Figure 2D). But, it is interestingly that the FITC-Hp1404 can penetrate the cell membrane of *S. aureus* and accumulate in the cytoplasm at low concentration (Figure 2B), which is similar to the linear α-helical peptide Buforin II [19]. As a short peptide with the length of 14 amino acid residues, Hp1404 may be via a floodgate mechanism to translocate to the cytoplasm [42]. As we know, after penetrating the membrane, AMPs can inhibit the macromolecular biosynthesis or interact with specific vital components inside the microorganisms (43, 46, 47). Thus, Hp1404 may have an unknown intracellular target, which needs further study to be confirmed in the future. On the other hand, Hp1404 may interact with the membrane and lead to the lateral expansion of the membrane area at high MIC concentration, which result in forming mesosome-like structures (Figure 2C-c), and lead to cell lysis (Figure 2C-d). It is the same as PGLa [40]. Due to the complex mechanism of action, it is difficult for *S. aureus* to develop resistance to Hp1404 (Figure 3), compared to conventional antibiotics.

Although AMPs have advantages over conventional antibiotics, there remain obstacles that prevent their therapeutic use: (i) Most AMPs, especially the α-helical AMPs, are toxic to mammalian cells due to less selectivity for target cells. For example, the MICs of bee venom peptide melittin against bacteria are 0.2-2 µM, while it has a CC_50_ of 0.7 µM, and has a HC_50_ of 0.51 µM [43], [44]. (ii) The loss of antimicrobial activity under physiological conditions or in serum also hampers the clinical applications of AMPs [45], [46]. There is a significant loss of *in vitro* activity of hBD-1 as the salt concentration increased from 50 mM to 125 mM [47], and the activity of LL-37 was completely suppressed in human serum [48]. In this study, it was found that Hp1404 has a low toxicity toward hRBC (HC_50_: 146.5µM) and human cell lines (10–30% toxicity at 64.6 µM). Moreover, the MICs of Hp1404 were tested under the salt concentration of about 170 mM, and Hp1404 still had *in vivo* effect under the normal physiological condition (Figure 5). However, our further experiments showed that serum decreased the activity of Hp1404 (data not shown), which may be the reason why the one dose of Hp1404 at 10 mg/Kg injected by i.v. did not cure all the MRSA P1381 infected mice (Figure 5B). Although Hp1404 does not have a good stability in serum, its low toxicity toward both mammalian cells and mice and non-inducing drug resistance suggest that it has the potential for applications.

In summary, Hp1404 is a cationic AMP from the scorpion *H. petersii*, and has a specific activity against gram-positive bacteria including MRSA. Hp1404 can penetrate the membrane of *S. aureus* at low MIC concentration, and can also interact with the membrane and result in forming mesosome-like structures at high MIC concentration. Moreover, *S. aureus* does not develop drug resistance after multiple treatments with Hp1404. Furthermore, Hp1404 has low toxicities to both mammalian cells and mice at its effective antibacterial concentrations, and can effectively protect mice from the infection of MRSA. Thus, Hp1404 may have potential applications as an antibacterial agent.
